# Testosterone, Endothelial Health, and Erectile Function

**DOI:** 10.5402/2011/839149

**Published:** 2011-09-06

**Authors:** Angela Castela, Pedro Vendeira, Carla Costa

**Affiliations:** ^1^Institute for Molecular and Cell Biology of the University of Porto (IBMC-UP), Rua do Campo Alegre, 823, 4150-180 Porto, Portugal; ^2^Department of Biochemistry (U38-FCT), Faculty of Medicine of the University of Porto, Alameda Professor Hernani Monteiro, 4200-319 Porto, Portugal; ^3^Department of Urology, Hospital de S. João, Alameda Professor Hernani Monteiro, 4200-319 Porto, Portugal; ^4^Department of Experimental Biology, Faculty of Medicine of the University of Porto, Alameda Professor Hernani Monteiro, 4200-319 Porto, Portugal

## Abstract

Experimental and clinical studies have reported that testosterone has a critical role in the maintenance of homeostatic and morphologic corpus cavernosum components, essential for normal erectile physiology. Although the exact mechanisms mediated by testosterone in erectile function are still under investigation, recent research has suggested an important role in the regulation of endothelial cell (EC) biological functions. Besides stimulating the production of EC mediators, testosterone is also thought to promote the vasculogenic reendothelialization process, mediated by bone marrow-derived endothelial progenitor cells. Additionally, testosterone seems to modulate other erectile tissue components, including trabecular smooth muscle cells, nerve fibers, and tunica albuginea structure, all essential for the erectile process. This paper summarizes current data regarding testosterone-induced cellular and molecular mechanisms that regulate penile tissue components, focusing particularly on the role of testosterone in endothelial health and erectile function.

## 1. Erectile Function: Intracellular Signaling Mechanisms

Erectile function is a complex neurovascular physiological mechanism that depends on the interplay between central and peripheral nervous system, vascular endothelium, and hormonal factors, as well as, the structural integrity of the cellular components of the penis. Erectile tissue is composed of small resistance helicine arteries that empty into sinusoidal spaces lined by a monolayer of vascular endothelial cells (ECs), embedded in a meshwork of interconnected smooth muscle cells (SMCs), and extracellular matrix formed by collagen, elastic fibers, and fibroblasts [1–3]. The complex interaction of this molecular and histomorphological system is crucial for the hemodynamic and mechanical processes involved in normal erectile function. During erectogenesis, a sexual stimulus results in parasympathetic nonadrenergic noncholinergic cavernosal nerves activation, leading to the production of neural nitric oxide (nNO) by neuronal NO synthase (nNOS) [4]. nNO diffuses into adjacent SMCs and activates the enzyme guanylyl cyclase, resulting in the generation and intracellular increase of cycling guanosine monophosphate (cGMP). This mediator activates multiple signaling cascades, leading to a decrease in intracellular Ca^2+^ uptake, which eventuates in SMCs relaxation, cavernous sinusoidal dilatation, and NO-mediated inflow of arterial blood [5, 6]. This initial inflow of blood into the helicine arteries increases shear stress, a phenomenon that mechanically stimulates endothelial NOS (eNOS) to produce eNO, and the release of prostanoids and endothelium-derived hyperpolarizing factors (EDHFs) [7]. These result in the engorgement of sinusoidal spaces by compression of the subtunical venules, which allows the complete occlusion of penile venous outflow. This mechanism known as veno-occlusion allows the maintenance of an erection. This NO/cGMP-erectile-dependent mechanism is regulated by the action of phosphodiesterase type 5 (PDE5), an enzyme which selectively metabolizes cGMP, resulting in the loss of arteriolar dilatation and penile detumescence. Therefore, as PDE5 is critical for the intracellular regulation of cGMP levels, this enzyme is the target molecule for the action of PDE5 inhibitors (PDE5I), the first line pharmacological therapy currently used for the treatment of erectile dysfunction (ED) [8]. Thus, this complex endothelium-dependent neurovascular process plays an important role in erection, and any functional and/or structural alterations in corpus cavernosum (CC) components may impair vasorelaxation and veno-occlusive mechanisms, compromising the initiation and maintenance of an erection, contributing to the development and progression of ED [9].

## 2. The Vascular Etiology of ED

ED is defined as the persistent inability to achieve and/or maintain a penile erection sufficient for satisfactory sexual intercourse [10]. Although the pathophysiology of ED is multifactorial, it has been mostly recognized as a disease of vascular origin. Thus, the loss of functional biology and structural integrity of penile endothelium, a condition designated as endothelial dysfunction (EDys), plays a pivotal role in the development of ED [11]. EDys is mostly induced by unifying processes, including alterations in the bioactivity/bioavailability of eNOS/eNO and increased oxidative stress in the penile vasculature. As result, eNOS-dependent vasodilatation is impaired and late structural vascular abnormalities may occur. In addition, there are changes in anticoagulation and anti-inflammatory processes, elevated leukocyte adhesion, and stimulation of SMCs growth, central mechanisms involved in both atherogenesis and ED [12]. In fact, it has been described that men with penile vascular dysfunction have also EDys in other vascular beds. Interestingly, the small diameter of the cavernosal arteries and the high content of endothelium (compared with other organs) suggest that the penile vasculature may be a sensitive indicator of systemic vascular disease [12]. Indeed, ED is currently considered as an early sign of subclinical EDys, occurring prior to the manifestation of cardiovascular disease (CVD). In fact, several vascular risk factors (VRFs), such as diabetes mellitus (DM), hypertension, obesity, lipidic disorders (a cluster of comorbidities defined as Metabolic Syndrome, MeS), and aging, are general correlates for both ED and CVD, with EDys being the common denominator [13–16]. Therefore, the above-mentioned VRFs are responsible for the deregulation of several metabolic and biochemical mechanisms involved in the disruption of endothelial functionality, contributing to both penile and generalized vasculopathy [16]. Additionally, the degree of EDys and vascular injury depends on the balance between endothelial damage, induced by exposure to VRFs, and the endogenous activation of repair processes. One of the mechanisms activated to regenerate the vasculature is angiogenesis, the process by which neighboring ECs are stimulated to proliferate and migrate, repairing the affected endothelial monolayer [17]. Furthermore, compelling studies have revealed that adult vasculogenesis also plays a critical role in the reendothelialization of damaged vasculature [18, 19]. When injuries occur in the endothelium, bone marrow- (BM-) derived endothelial progenitor cells (EPCs) are mobilized to the peripheral circulation and recruited to sites of vascular insult, where they differentiate into mature ECs integrating the vasculature [20]. Although EPCs biological activities are still under investigation, it has been suggested that mobilization, recruitment, differentiation, and integration in injured vascular sites could be impaired/affected by the chronic exposure to VRFs [21, 22]. In fact, recent studies have showed that patients with ED and CVD have reduced circulating levels of EPCs [23, 24], establishing a link between EDys and the aforementioned pathologies. Indeed, recent evidence suggested that the severity of EDys in penile tissue of patients with VRFs correlates with the imbalance between EDys and the impairment of endogenous repair processes, leading to alterations in essential events involved in normal erectile functionality [25].

## 3. EDys Links Testosterone Deficiency, MeS, and ED

Hypogonadism is a clinical and biochemical syndrome characterized by androgen deficiency and low serum testosterone levels. It can be classified as primary, testicular failure with elevated luteinizing hormone (LH), or secondary, hypothalamic-pituitary failure with decreased LH [26, 27]. Symptoms of hypogonadism include impaired libido, ED, difficulty in achieving orgasm, diminished sexual penile sensation, as well as, other physical disorders including fatigue, lack of physical strength, impaired cognitive function, and depressed mood [28]. It is well established that hypogonadism and ED are the most common disorders observed in the aging male, a condition designated as late-onset hypogonadism or androgen deficiency in the aging male (ADAM) [29]. In addition, a testosterone decline was also associated with chronic medical diseases such as, MeS, DM, and CVD, conditions with an increased morbidity and mortality [30]. Several epidemiological and interventional studies reported an inverse relationship between endogenous testosterone and increased VRFs, and overall mortality. Low testosterone levels were positively associated with the presence and severity of atherosclerosis and may contribute to increased arterial stiffness and to other marked effects on the cardiovascular system (i.e., coronary heart disease, myocardial infarction, and sudden cardiac death) [31–33]. Interestingly, studies revealed that men with established CVD often have low testosterone levels, which are associated with certain degrees of EDys, independently of the presence of other VRFs. Additionally, replacement testosterone therapy may have a beneficial impact by slowing the progression of CVD, and also by preventing ED and ameliorating endothelial function, suggesting a protective effect of endogenous testosterone on the endothelium [32, 34]. Furthermore, clinical and experimental observations also suggest that reduced testosterone levels may exacerbate some of the co-morbidities associated with the MeS. Indeed, low concentrations of testosterone in men have been associated with type 2 DM (T2DM), visceral obesity, insulin resistance, hyperinsulinemia, dyslipidemia, and increased deposition of abdominal adipose tissue [34–36]. In addition, testosterone replacement therapy significantly improved lipid profiles, reduced body fat, lowered blood pressure, decreased glucose levels, and improved insulin sensitivity in hypogonadal men with DM [35, 37, 38]. Similarly, correction of testosterone levels in obese men was shown to reduce body mass index, visceral fat mass, hyperlipidemia, and improved insulin sensitivity [35, 39, 40]. In other studies, testosterone treatment for ED ameliorated MeS components and improved endothelial function [41, 42]. Additionally, it was demonstrated that in hypogonadal men with ED and venous leakage, administration of testosterone therapy alone ameliorated erectile function by improving veno-occlusion [43, 44]. Testosterone may have a beneficial effect on the systemic vasculature, by ameliorating EDys, reinforcing the idea that testosterone supplementation could prevent and/or delay the progression of MeS, DM, CVD, and ED.

## 4. Testosterone, Endothelial Health, and Erection

EDys is an early event in the development of vascular diseases that later become clinically overt. Additionally, ED is currently considered as a very early warning sign of EDys and a silent indicator of a more generalized vascular systemic disorder. Impairment of EC structure and functionality at penile and systemic level has been described in both experimental and clinical studies and related to low circulating testosterone levels. Studies revealed that mature ECs express androgen-receptors, suggesting that testosterone may have a direct genomic-mediated action through the classic activation of nuclear androgen receptors [45]. Additionally, testosterone may promote a nongenomic effect potentially induced directly in the absence of a receptor, through a nontranscriptional effect, or by a distinct nonclassical receptor that is possibly associated with the plasma membrane [46]. It was reported that testosterone deprivation in rats, submitted to castration or to 5*α*-reductase inhibitor treatment (inhibits the conversion of testosterone to dihydrotestosterone), produced injuries in aortic ECs detected by electron microscopy [47]. ECs ultrastructure in orchidectomized animals seemed severely crimpled, coarse, and protuberant; with ruined cell-cell connections, and adhesion of red blood cells to the surface. Interestingly, administration of testosterone to these animals showed some degree of endothelial structural recovery, with few noticeable lesions detected in the monolayer [47]. In addition, a clinical study in male outpatients who underwent measurements of endothelial systemic function by brachial artery flow-mediated dilatation (FMD) with ultrasonography demonstrated that the levels of testosterone directly correlated with the percentage of FMD [32]. This report suggested that low plasma testosterone levels were associated with EDys in men, independently of the presence of other VRFs, corroborating the idea that testosterone has a protective effect in endothelial functionality [32]. At penile level, studies in animal models suggested that testosterone deficiency is associated to apoptosis of cavernosal vascular ECs [48] and that testosterone replacement therapy was able to induce CC cell proliferation and endothelial DNA synthesis [49]. Additionally, testosterone has been suggested to modulate several pathways on different erectile tissue components crucial for normal erection.

### 4.1. Modulation of NO and PDE5 by Testosterone

Recent publications have attempted to describe and clarify the role of testosterone in the regulation of molecular signaling pathways and cellular events in penile tissue, contributing to the maintenance of structural homeostasis. Indeed, several clinical and experimental studies revealed that deprivation/deficiency of testosterone may induce decrease in NO-mediated muscle relaxation, due to a reduction in the expression/activity of NOS; SMCs apoptosis; adipose tissue deposition with associated fibrosis of the CC; abnormal fibrosis in the tunica albuginea; impaired neural supply and disruption of the endothelial integrity and functionality [50–52]. As aforementioned, the critical role of the NO/cGMP signaling pathway on penile vasorelaxation events and erectile function is well documented. Compelling evidence suggests that testosterone is involved in the regulation of corporeal expression/activity of NOS isoforms (eNOS and nNOS), thus maintaining an adequate NO supply. Studies performed in orchidectomized animals demonstrated that administration of testosterone or its metabolite 5*α*-dihydrotestosterone restored erectile responses and NOS bioactivity, increasing NO production in CC and penile arteries [53–55]. In addition, molecular studies showed that penile tissue of castrated animals has reduced nNOS mRNA transcripts compared to control animals [56], corroborating the role of testosterone in stimulating nNOS expression. However, it still remains to fully elucidate the exact molecular basis underlying the regulation of NOS by testosterone. Besides increasing NO-mediated vasorelaxation essential for erectogenesis, testosterone plays also a paradoxal role in the modulation of PDE5 expression and activity. As aforesaid, PDE5 is the enzyme responsible for catalyzing cGMP into GMP, restoring SMCs contractility and penile flaccidity. Concordantly, disrupting PDE5 expression/bioactivity leads to alterations in normal erectile physiology. Castration in animal models has shown to reduce PDE5 levels/action in penile tissue. Conversely, testosterone treatment was shown to upregulate PDE5 gene and protein expression, as well as, the enzyme activity [57–59]. As PDE5 is the pharmacological target for PDE5I and its activity is reduced by hypogonadism, adequate levels of testosterone may be necessary to improve the therapeutic efficacy of PDE5I in the management of ED. Despite the evidence provided by studies in orchidectomized animals, the role of testosterone in human erectile function is still poorly understood. Clinical trials demonstrated that testosterone therapy in hypogonadal men with ED can increase the number and quality of erections in 40–60% of patients [60–62]. Nevertheless, other clinical studies demonstrated that combination therapy with PDE5I and testosterone in hypogonadal patients with ED significantly improves the ability to achieve and maintain an erection [63–65]. This reinforces the idea that testosterone replacement therapy should be considered for the treatment of ED in men with low testosterone levels who have previously failed to respond to PDE5I alone. In sum, testosterone seems to have a dual action in the modulation of the NO/cGMP signaling mechanism by: upregulating NOS expression (NO and consequently cGMP synthesis) and modulating PDE5 activity (cGMP degradation) in penile tissue. This may be seen as a paradox, since testosterone upregulates erection initiation molecules (NOS) and reduces the expression/action of PDE5 involved in penile detumescence. The testosterone-induced balance between these enzymes may suggest a role in the regulation of penile erection homeostatic mechanisms.

### 4.2. Beyond NO—Testosterone Modulates Cavernosal Proteins

It is known that vascular endothelium exerts regulatory functions on trabecular components, via the production of eNO and through the secretion of prostaglandins, endothelin (ET), platelet-derived growth factor, and transforming growth factor *β*1 (TGF-*β*1) [66–69]. Testosterone is thought to modulate corporeal properties by directly regulating the production of these molecules in penile ECs. Indeed, testosterone was reported to decrease the synthesis and release of the paracrine factors ET-1 and TGF-*β*1 and to reduce the expression of inflammatory markers [50, 51]. ET-1 is a potent vasoconstrictor secreted by ECs, which also exerts a mitogenic effect on fibroblasts and on vascular SMCs, stimulates matrix biosynthesis, and acts as a survival factor for myofibroblasts [70, 71]. Clinical studies have shown that hypogonadal men have increased levels of circulating ET-1, which almost decrease to normal concentrations after testosterone replacement treatment [72]. Additionally, TGF-*β*1 secreted by ECs was reported to stimulate collagen synthesis in both CC and tunica albuginea [73, 74]. In experimental models, expression of TGF-*β*1 in the penis is thought to be downregulated by the testosterone action [75]. Testosterone may also be directly involved in the reduction of inflammatory markers expressed by ECs, protecting vascular endothelial structure and function [50]. In fact, it was reported that incubation of ECs with testosterone or 5*α*-dihydrotestosterone can positively affect endothelial function through the reduction of the inflammatory response. It was suggested that the underlying mechanisms, involved the inhibition of tumor necrosis factor-alpha-induced activation of transcription factor-kappa B [76, 77]. Besides testosterone-mediated effects in corporeal ECs, modulating the expression of proteins that in an autocrine and paracrine way regulates CC function, it is also known that testosterone may directly regulate the structure and organization of all the other erectile tissue components. For instance, some publications have demonstrated that testosterone modulates the proliferation of trabecular SMCs and fibroblasts. Studies in orchidectomized animals showed that testosterone deprivation induces penile SMCs degeneration and apoptosis, with a significant increase in connective tissue deposition [58, 78, 79]. Concordantly, cavernosal SMCs counterpart was restored by testosterone replacement and erectile function reported to improve [58, 79]. Furthermore, ultrastructural analysis also demonstrated spatial trabecular SMCs disorganization with large number of cytoplasmatic vacuoles and decreased amount of myofilaments [52, 80, 81]. Moreover, animal models of testosterone deprivation presented penile tissue accumulation of adipocytes, particularly in the subtunical region, which may also contribute to corporeal-occlusive dysfunction [82]. Additionally, part of the vasorelaxation effects of testosterone was reported to be mediated through the nongenomic activation of penile SM adenosine triphosphate-sensitive K^+^ channels, in an endothelium-independent fashion [83]. Testosterone seems also essential for the maintenance of the tunica aluginea structure. In fact, the tunica albuginea of castrated animals was thinner and presented fewer elastic fibers and disorganized collagen structures [84]. These alterations may contribute to ED by impairing tunica albuginea roles in the veno-occlusive process. In addition, testosterone is also thought to regulate the structure and function of penile nerve fiber network, modulating the response to sexual stimulation. In fact, experimental studies showed that testosterone deprivation by castration altered cavernosal nerve structure and reduced the intracavernosal pressure [85–87]. These effects were reversed by testosterone supplementation [86, 87]. Overall, decreased testosterone seems to affect all erectile tissue components. These cellular changes, together with NO/cGMP signaling pathway impairment, contribute to modifications in vasorelaxation mechanisms, culminating in ED. Besides being associated with an impairment of penile molecular events leading to a reduced capacity of SMCs and ECs to vasorelaxate, testosterone deficiency was also suggested to affect endothelial repair and reendothelialization.

### 4.3. Testosterone and the Vasculogenic Repair Process

It has been reported that the degree of EDys is considered to be the balance between endothelial injury and the endogenous repair capacity. Reduced testosterone concentrations have been linked to the impairment of the vasculogenic reparative process mediated by EPCs. Recently, several studies revealed that testosterone levels may influence the number and function of circulating EPCs, affecting endothelial reendothelialization. In fact, it was documented that hypogonadal men have low number of circulating EPCs, which increase significantly after testosterone replacement therapy, suggesting that testosterone may play a role in the mechanisms of EPCs release from the BM [88, 89]. Since it was also reported that EPCs express androgen receptors [90], this suggests that testosterone may have a direct effect on EPCs functions [90, 91]. Reinforcing this hypothesis, it was reported that testosterone may promote *in vitro* EPCs migration, proliferation, and colony-formation, through a direct action on androgen receptors [89]. However, the mechanisms by which testosterone regulates *in vivo* EPCs actions are still under investigation.

## 5. Conclusions

Erectile function is a hemodynamic process, which involves penile blood inflow and suitable veno-occlusion occurring in an appropriate hormonal *milieu*. Since the penis is predominantly a vascular organ, it is established that vascular insufficiency is the most common etiology present in ED cases. Loss of EC function, a condition refereed as EDys, plays a critical role in the development of ED and systemic vascular disorders. Several VRFs, including the cluster of comorbidities which compose the MeS, are key correlates for the development of ED and generalized vasculopathy, having EDys as the common link. Additionally, an inversed relationship between the presence VRFs and decreased testosterone levels was reported, suggesting that testosterone deficiency may be associated with EDys, ED, and CVD. In fact, testosterone is thought to be an important mediator of cavernosal and systemic endothelial function, by regulating homeostatic mechanisms. More importantly, testosterone is involved in the modulation of CC vasorelaxation events, by regulating NOS and PDE5 expression and function. Additionally, testosterone may have direct actions in other penile components such as SMCs, nerve fibers, and tunica albuginea, regulating their structure and function. Furthermore, it was described that testosterone may also stimulate the vasculogenic repair process, promoting reendothelialization of the VRFs-induced EC monolayer lesions, ameliorating EDys and consequently penile and systemic vasculopathy. Although testosterone promotes endothelial health and has an important role in erection, further research is needed unveil all the mechanisms by which testosterone promotes its beneficial effects.
